# Secondary hemophagocytic syndrome after renal transplantation: two case-reports

**DOI:** 10.1590/2175-8239-JBN-2018-0246

**Published:** 2019-07-18

**Authors:** José Narciso, Beatriz de Oliveira Neri, Gilberto Loiola de Alencar Dantas, Lara de Holanda Jucá Silveira, Maria Luiza de Mattos Brito Oliveira Sales, Tainá Veras de Sandes Freitas, Ronaldo de Matos Esmeraldo

**Affiliations:** 1 Hospital Geral de Fortaleza, Setor de Transplantes, Fortaleza, CE, Brasil.; 2 Universidade Federal do Ceará, Departamento de Medicina Clínica, Fortaleza, CE, Brasil.

**Keywords:** Lymphohistiocytosis, Hemophagocytic, Immunoglobulins, Kidney Transplantation, Linfo-Histiocitose Hemofagocítica, Imunoglobulinas, Transplante de Rim

## Abstract

Hemophagocytic syndrome or hemophagocytic lymphohistiocytosis (HLH) is an infrequent and underdiagnosed condition caused by an overactive immune response, resulting in blood cells phagocytosis. After kidney transplantation (KTx), HLH is usually secondary (or reactive) to infectious and neoplastic processes and has a high mortality rate. No effective treatment is available for this condition. Usual procedures include detecting and treating the pathology triggering the immune system dysregulation, other than administration of intravenous human immunoglobulin (IVIG) and high doses of steroids, and plasmapheresis. The best protocol for maintenance immunosuppressive therapy is also unknown. This article presents two cases of post-KTx reactive HLH that underwent adjuvant IVIG treatment and obtained good clinical results. Despite the high morbidity and mortality associated with reactive HLH after KTx, the early and precise diagnosis and the administration of IVIG therapy along with the treatment of the triggering disease, was an effective strategy to control HLH.

## RESUMO

A síndrome hemofagocítica (SHF) ou linfo-histiocitose hemofagocítica é uma condição infrequente e subdiagnosticada que tem por base a ativação excessiva da resposta imune, resultando em fagocitose das células do sangue. Após o transplante renal (TxR), a SHF é habitualmente secundária (ou reativa) a processos infecciosos e neoplásicos, culminando em elevadas taxas de mortalidade. Não há evidências quanto ao tratamento ideal dessa condição. Além de investigação e tratamento da patologia desencadeante do processo de desregulação do sistema imune, há descrições do uso de imunoglobulina humana (IVIG), esteroides em altas doses e plasmaférese. Não há evidências quanto à melhor forma de delinear a imunossupressão de manutenção. Este artigo apresenta dois casos de SHF reativa pós-TxR que realizaram tratamento adjuvante com IVIG, obtendo bons resultados clínicos. Apesar da elevada morbimortalidade associada à SHF reativa após o TxR, o diagnóstico ágil e preciso, associado à instituição de terapia com IVIG adjuvante ao tratamento da doença desencadeante, foi uma estratégia eficaz em conter o processo.

## INTRODUCTION

Hemophagocytic lymphohistiocytosis (HLH) syndrome is a rare, underdiagnosed, and severe condition caused by an overactive immune response, resulting in phagocytosis of blood cells. Clinical manifestations include fever, hepatosplenomegaly, cytopenia, hyperferritinemia, hypertriglyceridemia, and hypofibrinogenemia.^1^^,^ 2 ^,^ 3 ^,^ 4 After kidney transplantation (KTx), HLH is usually associated with infectious (76%) and neoplastic (27%) processes, and excessive immunosuppression is a contributing factor.^7^^,^ 8 ^,^ 9 The use of intravenous human immunoglobulin (IVIG), high-dose corticosteroids, and plasmapheresis have been reported as complementary therapies to achieve inflammatory response control. This report presents two cases of reactive HLH post-KTx, which received IVIG as adjuvant therapy, obtaining a good clinical response.

## CASE REPORTS

### CASE 1

EGNB, a 41-year-old male with end-stage renal disease (ESRD) of unknown cause, was submitted to deceased donor KTx in 2010. He received antithymocyte globulin induction immunosuppression at a dose of 6 mg/kg of body weight and maintenance with tacrolimus and mycophenolate sodium, progressing uneventfully until hospital discharge. The patient was followed up in an outpatient clinic, and after 6 years, he presented with lymphopenia (1018 cells/mm^3^), which progressively worsen (682 cells/mm^3^), and was admitted after having daily fever (38-39ºC) and non-inflammatory watery diarrhea for 2 weeks, without weight loss. On physical examination, he was in good general condition, with skin and mucosal pallor, normal cardiopulmonary parameters, and palpable hepatosplenomegaly.

The laboratory investigation revealed pancytopenia, altered inflammatory markers, and kidney dysfunction (Table 1). Tests for HIV, hepatitis B and C virus, Epstein-Barr virus, parvovirus B19, and syphilis were negative. Abdominal tomography confirmed homogenous hepatosplenomegaly. Due to persistent fever of unknown cause and worsening of clinical and laboratory conditions, antibiotic therapy with piperacillin-tazobactam was initiated. Cultures collected at admission were negative. The myelogram was performed, which revealed a mild marrow hyperplasia, slight dypoiesis and hemophagocytosis, leading to the diagnosis of HLH secondary to the infectious condition in an unknown site.

**Table 1:** Laboratory exams

Case 1: EGNB							
Parameter	Reference value	Admission	Pre-IVIG (8° DOH*)	End of IVIG (11° DOH*)	72h post- IVIG (14° DOH*)	23° DOH	Discharge (30º DOH*)
Hemoglobin (g/dL)	12 - 13	8.4	6.7	6.9	7.9	6.2	9.8
Leucocytes (por mm^3^)	4500 - 11000	3515	2005	3557	8263	3274	4877
Platelets (/ mm^3^)	150000-450000	109200	46500	22860	35210	161200	158400
Creatinine (mg/dL)	0.6 - 1.2	2.2	3.3	4.1	3.6	2.7	1.6
Ferritin (ng/mL)	16 - 300	25754			48666	4476	
Fibrinogen (mg/dL)	180 - 350		146				
Triglycerides (mg/dl)	< 150		447				
TGO (U / L)	13 - 39	67	623	302	251	68	31
TGP (U / L)	7 - 52	92	472	314	173	91	52
Caso 2: GBSA							
Parameter	Reference value	Admission	Pre-IVIG (12° DOH*)	End of IVIG (19° DOH*)	72h post- IVIG (14° DOH*)	36° DOH*	Death (47º DOH*)
Hemoglobin (g/dL)	12 - 13	7.3	7.7	7.4	7.1	9.1	11.9
Leucocytes (/mm^3^)	4500-11000	7085	11980	9944	8067	9200	20600
Platelets (/ mm^3^)	150000-450000	108400	126400	51670	45550	88160	16000
Creatinine (mg/dL)	0.6 - 1.2	0.9	0.8	1.2	1.2	0.9	1.3
Ferritin (ng/mL)	16 - 300	4405	7157		4256		
Fibrinogen (mg/dL)	180 - 350		530		631		
Triglycerides (mg/dl)	< 150	518	349		312		
TGO (U / L)	13 - 39	234	101	75	44	18	204
TGP (U / L)	7 - 52	168	49	9	10	19	39

* DOH: Day of hospitalization.

Despite antibiotic therapy, the patient developed hypoxemic respiratory insufficiency and severe graft dysfunction due to septicemia, requiring invasive ventilatory support and hemodialysis therapy. Due to clinical severity, adjuvant therapy with intravenous IVIG (2 g/kg body weight, divided in 3 doses) was instituted, as well as reduction of immunosuppression maintenance therapy (mycophenolate suspension), and expansion of the antibiotic therapy by replacing piperacillin-tazobactam with meropenem, vancomycin, and liposomal amphotericin B. An improvement of clinical and laboratorial parameters was achieved (Table 1), and the patient was removed from hemodialysis therapy. He was discharged from hospital after one month of hospitalization and is on regular outpatient follow-up without further complications.

### CASE 2

GBSA, a female of 61 years old with ESRD due to hypertensive nephrosclerosis, underwent deceased donor KTx in December 2015. She received antithymocyte globulin induction immunosuppression at a total dose of 6 mg/kg body weight, 3 plasmapheresis sessions, and IVIG at total dose of 2 g/kg, as well as maintenance therapy with tacrolimus, everolimus, and prednisone, progressing without complications until hospital discharge.

About one year after KTx, she was admitted to the hospital with dry cough, low fever, hyporexia, asthenia, and polyarthralgia for 1 month; she had lost approximately 6 kilos in this period. Previous examinations revealed anemia and thrombocytopenia (Table 1). She was then hospitalized for evaluation, diagnosis, and clinical support.

**Figure 1:**
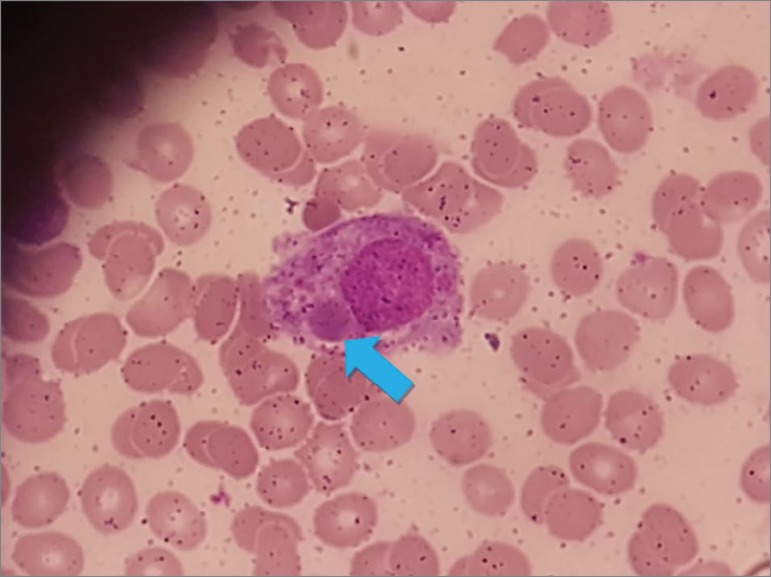
Bone marrow aspirate showing marked hemophagocytosis (case 2).

At admission, the patient was feverish, tachypneic, tachycardic, and pale. She had lung auscultation crackles in the lower right third and abdomen dullness to percussion over Traube’s space. Antibiotic therapy was started with levofloxacin after evaluation and suspected diagnosis of community-acquired pneumonia. Admission examinations revealed persistence of bicytopenia, in addition to hyperferritinemia and hypertriglyceridemia. Renal function was preserved. Total abdominal ultrasonography confirmed the presence of homogeneous splenomegaly. The patient followed with progressive clinical worsening (persistence of fever, tachycardia, and hypotension). Antibiotic therapy with piperacillin-tazobactam and later with meropenem was scaled-up due to unsatisfactory clinical response. Given the cytopenia, a myelogram was performed two weeks after admission, which revealed hypercellularity and hematopoiesis, increased macrophage activity, and bone marrow with reactive characteristics (Figure 1). HLH secondary to an unknown infectious process was diagnosed. Mycoculture was negative. Concomitantly, increasing levels of galactomannan in blood led to the initiation of antifungal therapy with voriconazole for aspergillosis. Therefore, IVIG at 2 g/kg body weight, divided into 3 doses, was initiated combined with the specific therapy, and immunosuppression was reduced (suspension of everolimus and maintenance of tacrolimus and prednisone).

After these measures, the patient clinical status improved significantly, with reduction of inflammatory markers and gradual improvement of hematological dysfunction (Table 1).

However, after 40 days of hospitalization, the patient had a massive pulmonary aspiration, clinical worsening, and death one week after the event.

## DISCUSSION

HLH is a high-mortality disorder (50% in primary HLH and 10-15% in secondary HLH), characterized by an exacerbated immune response with the release of proinflammatory cytokines, hyperactivation of macrophages in the reticuloendothelial system (liver, spleen, and bone marrow), resulting in intense hemophagocytosis.^1^^,^ 2 ^,^ 4 HLH was first described in 1952 by Farquhar and Claireaux as a hereditary medullary reticulosis.^5^ The first 19 cases were published in 1979 by Risdall et al., 13 of which occurred after renal transplantation,1 was due to SLE, and 5 were idiopathic.^4^^,^ 5 ^,^ 10 ^,^ 12 ^,^ 14 In 1985, Hadchouel reported the first HLH cases associated with rheumatic pathologies and named the disease macrophage activation syndrome.^3^^,^ 5 The condition is underdiagnosed, and should always be considered in cases of febrile cytopenia with hepatosplenomegaly. Delay in diagnosis leads to critical multi organ dysfunction and reduced survival rate (34% at 42 months).^7^

Primary HLH is of genetic origin, has an autosomal X-linked inheritance, is most common in childhood, its incidence varies considerably (1 to 1.2 in every 50,000 to 1 million live births) and has no sex predominance.^1^^,^ 2 ^,^ 5 It can be associated with immunodeficiencies, such as Chédiak-Higashi and Gricelli syndromes, as well as X-linked lymphoproliferative diseases. The genes involved are related to NK cells activation defects or their cytotoxic effect.^1^^,^ 2 ^,^ 3 ^,^ 7 ^,^ 8 ^,^ 9 ^,^ 11

Secondary HLH can occur at any age, more commonly in adults. The mean age is 50 years, with a sex ratio of 1.5 and 3.0 males for 1 female. Secondary HLH is associated with infections (viral, fungal, bacterial, and parasitic), drugs (methotrexate, non-hormonal anti-inflammatories, anticonvulsants, anti-TNF-α, anti-CD52), autoimmune diseases (systemic lupus erythematosus, rheumatoid arthritis, Sjögren syndrome, systemic sclerosis, and Kawasaki disease), neoplasms (lymphomas), and immunosuppression (solid organ transplantation).^1^^,^ 2 ^,^ 5 ^,^ 9 ^,^ 10 ^,^ 12 ^,^ 13 ^,^ 14 ^,^ 16 A diagnostic criteria was proposed in 1991 and revised in 2004 by the International Society of Histiocytosis and includes 8 items of which at least 5 must be present (Chart 1).^1^^,^ 2 ^,^ 6 ^,^ 8

**Chart 1:** Hemophagocytic lymphohistiocytosis criteria

Fever (> 38ºC for at least 7 days)
Cytopenia affecting 2 or 3 cell types (hemoglobin < 9.0g/dL; neutrophils < 1.000/mm^3^; platelets < 100.000/mm^3^)
Splenomegaly
Increased triglycerides (> 265 mg/dL) and/or hypofibrinogenemia (< 150 mg/dL)
Increased soluble CD25 (> 2.400 U/mL)
Reduced or absent natural killer cells activity
Evidence of hemophagocytosis (marrow, spleen, or lymph node)

The two case-reports described post-KTx HLHs secondary to infectious processes with at least 6 diagnostic criteria each. In both cases, there was intense immunosuppression. The first patient was on outpatient follow-up with progressive lymphopenia until hospitalization due to HLH. The second patient was of high immunological risk and had received prophylaxis for antibody-mediated induction therapy with antithymocyte globulin, in addition to plasmapheresis and IVIG.

The diagnosis of post-KTx HLH is a challenge, since patients frequently present misleading factors. As examples, pancytopenia and hypertriglyceridemia may be present as adverse events associated with immunosuppressive drugs.^10^ Another difficulty is the differential diagnosis with sepsis, delaying HLH diagnosis and worsening the outcome. Markers of worse prognosis include age over 30 years, absence of lymphadenopathy, marked pancytopenia, alkaline phosphatase, β2-microglobulin, and elevated bilirubin, as well as marked disseminated intravascular coagulation and hyperferritinemia.^5^

The overall treatment goal of post-KTx HLH is to control the excessive immune response and prevent multi organ damage by treating the triggering clinical condition.^7^ However, the severe and rapid disease progression often requires an adjuvant treatment to contain the inflammatory response, but no consensus is available about the best supportive therapy for HLH. Emmenegger et al. reported a 59% response with 1.6 g/kg IVIG for 3 days in patients with different underlying conditions (infection, malignancy, and systemic lupus erythematosus), confirming the effectiveness of this therapeutic strategy, especially with the infectious condition (78% versus 39%).^5^^,^ 10 ^,^ 12 ^,^ 15 ^,^ 17 The main predictive factor of good clinical response was starting therapy within 2 days of serum ferritin peak.^5^^,^ 15 Asci et al. reported that of the 13 patients with post-KTx HLH analyzed in their study, 6 recovered and received IVIG therapy.^10^^,^ 13 HLH was controlled in both cases of our study with IVIG as an adjuvant treatment to broad-spectrum antibiotic therapy and reduction of maintenance immunosuppressive therapy, without additional steroids.

As a limitation of this study, levels of soluble CD25, as well as the activity of natural killer cells could not be assessed. Those are useful markers for patients under suspicion of HLH, since they directly translate the imbalance between activation and control of the immune response. CD25 indicates the level of the interleukin-2 (pro-inflammatory) receptor, which has an impact on the proliferation of antigen-presenting cells, lymphocytes, and histiocytes.^8^ Natural killer cells activity indicates the immune cytotoxic capacity and, when reduced, the loss of proliferative control of inflammatory cells, which may also be influenced by the immunosuppression of patients.^2^^,^ 10

## CONCLUSION

Post-KTx reactive HLH is a condition of high morbidity and mortality, and the adjuvant therapy with IVIG seems to be an effective treatment strategy. Rapid diagnosis is crucial for the adequate control of HLH. In some cases, it is difficult to state whether immunosuppression itself led to the development of HLH or whether the immunosuppressive therapy favored the emergence of secondary processes that culminated in the inflammatory disorder. In the present cases, it is plausible that infections were the cause of the disorder because of the syndrome onset after KTx.
